# Book Reviews

**Published:** 1823-02

**Authors:** 


### CRITICAL ANALYSIS

OF

### ENGLISH AND FOREIGN LITERATURE

RELATIVE TO THE VARIOUS BRANCHES OF

### Medical Science.

Quæ laudanda forent, et quæ culpanda, vicissim

Illa, prius, cretâ; mox hæc, carbone, *notamus.*—PERSIUS.

### DIVISION I.

### ENGLISH.

ART. I.—*A Treatise on Dislocations, and on Fractures of the Joints.* By Sir ASTLEY COOPER, Bart. F.R.S. Surgeon to the King, &c. &c. &c.—1 vol. 4to. 30 plates, pp. 562. Longman and Co. London.

ON resuming our critical labours at the commencement of a new year, we may, perhaps, be permitted to say a few words relative to an occupation so useful, and yet so invidious,—so necessary to be done, and yet so difficult to do with satisfaction to the sensitive feelings of an author, or the rigid scrutiny of the indifferent or sceptical. That we have hit this nice point, we do not even dream of asserting; but we have assuredly endeavoured to do so: we have uniformly taken up the book to be reviewed, with a determination to forget, as far as possible, the author; to extract whatever we found to be useful, or new in theory or practice; to reprehend what we conceived to be erroneous in either, so that the profession at large, and the junior part of it in particular, should not be misled by false doctrines sanctioned by high authority: nor should the good be passed by, because it presented itself to us in an unassuming, or perhaps even in an homely, garb. Upon all occasions we have endeavoured to separate the author from his work: the latter is public property, the former is sacred: the work may, if faulty, be productive of incalculable mischief, and must be exposed; our duty to our readers demands it, and, as far as our judgment and abilities permit, this object it shall always be our aim to accomplish. This intellectual dissection we will endeavour to perform in as cleanly and decent a manner as we can; for, in this, as in other dissections, the design is not to expose the subject, but to instruct the lookers on.

From this short digression we turn to the splendid work before us; in bulk, in beauty of paper and type, in the number of plates, and in the excellence of their execution, surpassing any modern publication on a professional subject with which we are acquainted,—at least in this country. On turning over the pages of this book, we were at first tempted to exclaim against the numerous errata of the press which we encountered on every side; but, when we began the perusal *seriatim*, we were so struck with the author's candid avowal of this error, that we were induced to strike out the reprehension which this unlucky discovery had elicited. However, we must notice particularly one of these errata in this place, because it is not corrected, and it directly affects the sense of the passage: it occurs in page 338, and will be more particularly pointed out in its place. Our experienced author also endeavours to obviate any objection that may be raised against the familiar and occasionally colloquial style of the work; declaring "that he had rather be seen in a good plain suit, than in the finest embroidered dress." (Preface, p. vii.) It is, however, to be recollected that a certain attention to dress is necessary to obtain admission into the *best society*; and (dropping the metaphor) that, as this volume is intended to go down to posterity, and will most assuredly do so, the style is a matter of more importance than in those ephemeral publications which are born only to die.

Many may consider these preliminary remarks, perhaps, as rather fastidious; but let them recollect that Sir Astley Cooper is likely to be quoted as an authority, and followed as an example; and, therefore, it behoves us more especially to notice those points in which he has failed, lest they should be adopted by others who do not possess his eminent and redeeming merits.

We have but one more remark to make before we enter upon the analysis of the work, and that remark relates to the price at which this book has been published: certainly a very noble instance of liberality on the part of the author, but which must not lead us to form unfair conclusions with respect to other authors less fortunately circumstanced, and who have it not in their power, whatever their wishes might suggest, to follow this splendid example.

The bulk of this volume consists of a reprint of the Essays on Dislocation, published in the octavo edition, with some additional matter: the plates, however, are new, and increased both in number and size, as well as in beauty of execution. Sir Astley Cooper informs those who are in possession of the former edition, that, for their convenience, he will print the additional matter in the octavo form, provided they express their wishes and send their names and address to him, within three months after this publication.

Sir A. Cooper commences his work with remarks on dislocations in general, and almost immediately details an interview which a patient, whose shoulder had been dislocated "many weeks," had with him. It appears that the surgeon in the country had mistaken the nature of the accident; and our author's advice to the patient was, not to suffer any attempt at reduction. We do not in the least doubt that Sir A. Cooper's advice was highly judicious; but he seems to anticipate, in his preface, that his professional brethren may imagine that he has limited the period at which reduction may be attempted too strictly: and, with respect to dislocations of the shoulder, there is some reason to think that he has. " A considerable share of anatomical knowledge" (we quote Sir Astley's words,) " is required to detect the nature of these accidents, as well as to suggest the best means of reduction; and it is much to be lamented that students neglect to inform themselves sufficiently of the structure of the joints. They often dissect the muscles of a limb with great neatness and minuteness, and then throw it away *without any examination of the ligaments; a knowledge of which, in a surgical point of view, is of infinitely greater importance.*" We have printed this very important remark in italics, in order to call the particular attention of our younger readers to it, fully agreeing with our author, both in the truth of his remark, and great importance of impressing it strongly on the minds of all classes of students in our profession.

Yet, with the most accurate knowledge of the structure of the joints, the tumefaction and tension arising from the injury occasionally so obscure the nature of the accident as to render it extremely difficult to be detected; therefore, conclusions drawn when the swelling has subsided, the muscles are wasted, and the head of the bone can be distinctly felt, would be both " illiberal and unjust."

The immediate effect of a dislocation is to alter the form of the joint; often to produce a change in the length of the limb, to occasion the almost entire loss of motion, and to alter the axis of the limb. In the first moments of the dislocation, it is to be remembered that considerable motion remains. In a case at Guy's Hospital, where the thigh was dislocated into the foramen ovale, a great degree of mobility of the bone existed at the dislocated part, but in less than three hours it became firmly fixed in its new situation by the permanent contraction of the muscles. This is very important to remember, because mobility of the bone is one of the most marked symptoms of a fracture of its neck, though in this case the knee is turned *outwards.* After describing the usual criteria by which dislocations are known, Sir Astley observes that, among the more remote effects of these accidents, the crepitus produced by the effusion of adhesive matter into the joint and bursæ, may induce the practitioner, if he be not aware of it, to suspect a fracture where none has occurred. Inflammation of the joint occasionally is also so severe as to produce suppuration, and to destroy the patient, even after the reduction of the dislocation; and two cases of fatal result are mentioned. (p. 7.) We believe this seldom occurs, except in dislocations of the thigh.

Sir Astley's description of the dissection of dislocated joints is, of course, accurate; but, as it presents no novelty, we pass on to observe, that dislocation sometimes arises merely from a relaxation of the ligaments of the joints, of which three instances are inserted, where the patella was dislocated in that manner. Relaxation or paralysis of the muscles will also sometimes produce the same effect; but these accidents may be considered as comparatively rare.

It is well known that the hip-joint frequently becomes dislocated in consequence of ulceration. Sir Astley Cooper mentions a preparation, now at St. Thomas's Hospital, where the knee was dislocated by ulceration; and a case of the same kind occurred in a boy, a patient at Guy's Hospital.

Dislocation accompanied with fracture is a common occurrence at the ankle-joint. At the hip-joint, the acetabulum is occasionally broken off. The head of the humerus, and the coronoid process of the ulna, also may experience the same fate.

In the event of a fracture and dislocation occurring at the same time, our author advises the dislocation to be reduced before the fracture be adjusted, and confirms his opinion by the case of a gentleman who had his leg broken and his shoulder dislocated: the latter was not attempted to be reduced until a fortnight after the accident, and then the attempt failed, the fractured leg prohibiting the employment of the necessary degree of force.

A compound dislocation is next defined, its essence consisting in the exposure of the cavity of the joint, in addition to the displacement of the articulatory surfaces: of course, the effect is an extravasation of blood into the joint, and the escape of the synovia. (p. 18.) We need hardly add, that these accidents are declared by our author to be attended with *great danger*. In explaining the causes of this danger, Sir Astley says, "When a joint is opened, inflammation of the lacerated ligaments and synovial membrane speedily succeeds; in a few hours suppuration begins, and granulations arise from the synovial membrane, which, being a *mucous membrane*, is more disposed to the suppurative than to the adhesive inflammation." Here we humbly conceive there is a slight pathological error: we always regarded the essential characteristic of a mucous membrane to be, one which communicated with some external opening of the body; and we have been taught to consider the synovial membranes as a class by themselves. The leading circumstances that render this species of dislocation so serious are then described; but the mode of treatment is deferred until the compound dislocations of the ankle are described, "where they will be better understood; and thus a repetition, which would be both irksome and useless to the reader, will be avoided." (p. 19.)

On the causes of dislocation, our author observes, that, when the muscles are unprepared for resistances, very slight accidents will often bring about the effect. A fall in walking will sometimes dislocate the hip-joint, when the muscles have been prepared for a different exertion.

Dislocations of the elbow-joint in children, Sir Astley thinks to be rare; such cases usually being, in reality, fractures of the condyles of the os humeri, which assume the appearance of dislocation in consequence of the radius and ulna being drawn back with the fractured condyle.

In enumerating the circumstances that impede reduction, our learned author mentions the form of particular bones, or the cavity that receives them, may in part occasion the difficulty. He very judiciously combats the supposition that the capsular ligaments resist reduction; neither do they appear to have any power in preventing the occurrence of the dislocation: it is the ligaments peculiar to the joints, and the tendons spread over them, that form the principal obstacles to the displacement of bones, and the resistance of the muscles which is the most formidable obstacle to their re-adjustment. When a bone has been a long time displaced, the extremity also contracts adhesions to the surrounding parts. Sometimes also the socket of the bone becomes filled with adhesive matter; and, "lastly, a new bony socket is sometimes formed, in which the head of the bone is so completely confined that nothing but its fracture could allow it to escape from its new situation." (p. 29.)

The means of reduction are divided by our author into constitutional and mechanical: the former are principally three,—bleeding, warm-bath, and nausea. Of these, Sir Astley considers bleeding the most powerful; and the operation should be performed in the erect position, in order that syncope may the more speedily be induced: an opinion in which we entirely concur, and which we are surprised to find so often mentioned by different professional men, and yet so seldom practised. The warm-bath is next recommended, the method of using which is sufficiently known; and the third plan is that of exciting nausea, by means of tartarized antimony in small doses, which however will seldom succeed alone, as the operation of that medicine is so very uncertain and dissimilar in different individuals; and it becomes a matter of great difficulty to obtain the exact effect that we wish to produce. Our author, therefore, is induced to employ it chiefly to keep up the state of syncope already produced by either of the former methods.

In describing the mechanical means, it is observed that force must only be gradually employed; an excellent rule, too often neglected: for violent force most assuredly calls "up all the powers of resistance to oppose the efforts making by the surgeon."

The next precept is also of the highest importance: it impresses upon the surgeon the necessity of fixing the bone in which the socket is placed; a point which Mr. Bromfield has most ably stated and illustrated in his surgical works.

In dislocations of the hip-joint, pullies should always be employed; as also in those of the shoulder which have long remained unreduced. In attempting reduction, a relaxation of the principal muscles of the limb must be obtained by such a position as will best effect that object.

The following rule is also important, and concludes this branch of the subject: "Great advantage is derived in the reduction of dislocations from attending to the patient's mind; the muscles opposing the efforts of the surgeon, by acting in obedience to the will, may have that action suspended, by directing the mind to other muscles." (p. 34.)

Sir Astley concludes these general remarks by giving it as his opinion, that attempts at the reduction of the shoulder should not be made later than three months after the accident, and for that of the hip not after eight weeks: at the same time he is aware that the shoulder has been reduced at a much later period, though without any improvement to the patient, as far as the use of the limb was concerned.

We come now to the consideration of particular dislocations, and first in order is dislocation of the Hip-joint. This bone may be displaced in four different ways: 1st, upwards, or on the dorsum of the ilium; 2dly, downwards, or into the foramen ovale; 3d, backwards and upwards, or into the ischiatic notch; and 4thly, forwards and upwards, or upon the body of the pubes.

The dislocation upwards is the most common of these accidents: in this case, the limb is *shorter*, the knee and foot are turned *inwards*, the thigh cannot be separated from the other; the head of the bone may sometimes be perceived moving upon the dorsum of the ilium; the trochanter is less prominent, and the roundness of the hip is lessened, compared with the opposite side. In order that the surgeon may not confound this accident with the fracture of the neck of the bone, he must recollect that, in this latter case, the knee and foot are *generally* turned outwards, the trochanter is drawn upwards, the thigh can be bent *towards the abdomen*, and the limb, though shortened, can, by a little extension, be rendered of the same length as the sound one; sometimes also, in rotating the limb, a crepitus can be felt. This fracture (within the capsular ligament,) also seldom occurs but in aged subjects. In the dislocation upwards, the glutei and triceps muscles principally resist reduction.

In the description of the method of reduction, the leading points insisted upon are the gradual extension, the gentle rotation of the knee and foot, when the extension has been carried far enough; and the necessity that sometimes occurs of lifting the head of the bone over the lip of the acetabulum, which may be effected by placing an arm under the limb, near the joint, or by a napkin placed under it, and raised by an assistant. It is needless to add, that the extension in these cases must be made by means of pulleys, and the constitutional means recommended above must be previously employed. Thirteen cases are subjoined to illustrate the precepts above mentioned. It is to be observed, that, in many cases, particularly those of long standing, the bone returns into its socket without any snapping noise.

*On the dislocation downwards.*—This happens when the thighs are widely separated. In contradiction to what is usually said, Sir Astley Cooper asserts that, in this accident, the ligamentum seres is torn through; the thighs, and consequently that ligament, being upon the stretch at the time of its occurrence. The limb is shorter than the other in these cases. In very thin persons, the head of the bone may be felt upon the inner and upper part of the thigh towards the perinæum; the body is bent forwards; if the body be erect, the knee is considerably advanced; it is widely separated from the other. The foot is not generally turned either outwards or inwards, though in this respect it varies a little; and, finally, there is a hollow below Poupart's ligament.

The reduction of this accident is, says our author, very easily effected. If it has happened recently, place the patient on his back, separate the thighs as much as possible, and fix the body by placing a girth between the pudendum and upper part of the thigh, fixing it to a staple in the wall. The surgeon then puts his hand upon the ankle of the dislocated side, and draws it over the sound leg, and the head of the bone slips into the socket. (p. 67.) This plan, however, will not succeed if the dislocation has existed two or three weeks: in that case the pulleys are required; the thigh is to be drawn upwards, whilst the knee and foot are pressed down, to prevent the lower part of the limb being drawn with the thigh-bone. Great care must be taken not to advance the leg in any considerable degree, or the head of the thigh-bone may be forced into the ischiatic notch.

One case only of this accident is recorded; and the peculiarities of the limb are minutely described in another instance, which had not been reduced.

*On the dislocation backwards, or into the ischiatic notch.*—The anatomical description of the parts clearly shows that the direction of this dislocation is a little *upwards* as well as backwards; the head of the bone rests, in these cases, on the pyriformis muscle behind the acetabulum. This is the most difficult to detect, or to reduce. It seldom happens that the limb, in this dislocation, is more than half an inch shorter than its fellow; the head of the bone can seldom be distinctly felt; the knee and the foot are turned a little inwards, and the toe rests against the ball of the great toe of the other foot; the heel, when the patient is standing, does not quite reach the ground; flexion and rotation are in a great degree prevented. A description of a dissection, and an accompanying plate, explain the wonderful provisions of nature in adapting herself to new circumstances. This accident is caused by the thigh being bent at right angles on the abdomen, or *vice versâ*, and force applied to the knee pressing it inwards. The reduction is difficult. The usual mode of fixing the pelvis is followed; the thigh is brought across the middle of the sound one, and extension is then made; but, as it is necessary also to lift the bone over the lip of the acetabulum, an assistant passes a round towel under the upper part of the thigh and over his own shoulders, who, pressing with his hands upon the brim of the pelvis, lifts the bone by raising his body. (p. 78.) In the first case, a patient of Mr. Lucas's, we find that extension was made by pulleys in a right line with the body, the trochanter was thrust forward with the hand, and in two minutes the bone returned into its socket with a violent snap. The young surgeon will do well to recollect these two very different methods of arriving at the same end. The fifth and last case of this kind detailed is important, because it shows that the bone is occasionally reduced without any snapping or noise, so that the surgeon has nothing to trust to but the appearance and mobility of the limb to assure him that the bone has been properly replaced.

*Of the dislocation on the pubis.*—This accident is easy of detection: the limb is an inch shorter than the other; the knee and foot are turned outwards, and cannot be rotated inwards; the head of the bone may be distinctly felt on the pubis, above the level of Poupart's ligament, on the *outer side* of the femoral artery and vein. Notwithstanding these striking marks, Sir Astley Cooper has known three instances in which this accident was overlooked. In reducing this dislocation, the difference to be observed is, that the extension is to be made in a line *behind* the axis of the body, the thigh-bone being drawn backwards.

After extension has been carried on for some time, an assistant, with a napkin passed under the thigh, lifts the head of the bone over the pubes and edge of the acetabulum, pressing at the same time with one hand on the pelvis. Of twenty dislocations of the thigh, Sir Astley thinks the relative proportion would be, twelve on the dorsum ilii, five in the ischiatic notch, two in the foramen ovale, and one on the pubes.

We were surprised to find it asserted, upon the authority of Mr. CLINE, that SHARP did not believe that a dislocation of the thigh-bone ever occurred. Mr. Cline's authority no one can doubt; and, granting the fact to be so, we can only lament how much surgery must have retrograded from the days of honest WISEMAN, who has a short chapter on this very accident, which he says may happen in *four different ways.* This much, at least, is quite certain, that Mr. Sharp does not expressly treat of, or mention, dislocations of any kind, either in his Critical Inquiry, or in his Treatise on the Operations of Surgery.

It will be seen that we have closely analyzed the whole of Sir Astley's valuable observations on the subject of dislocations of the hip, conceiving that, by condensing the more important facts necessary to be borne in mind relative to these accidents, we shall have done an essential service to those who have not the means of getting immediate access to the work itself; so that, in the event of a sudden emergency, the practitioner might turn to our account, and not turn to it in vain. We know no form of compliment that can more substantially mark our estimation of the importance of the practical precepts it contains.

We come now to the consideration of Fractures of the Os Innominatum, which may be mistaken for dislocation, as the leg is somewhat shorter, the trochanter is more forward, and the knee and foot are turned inwards, as is the case in dislocation in the ischiatic notch; but it is to be remembered that, if the hand be placed on the crista of the ilium, and the thigh be moved, a crepitus may generally be felt; and there is more motion preserved than in dislocations.

*Of Fractures of the upper part of the Thigh-bone.*—Every surgeon knows the conflicting opinions that have been entertained with regard to the final result of these accidents. Sir Astley Cooper thinks that this difference of opinion has arisen from confounding three very different species of fracture under the indiscriminate appellation of fracture of the neck of the thigh-bone, (p. 115.) That these fractures admit of union our author admits, as far as concerns two species of them, but not with respect to the third. On this subject Sir Astley produces his observations on living subjects, the result of his dissections, and his experiments upon animals. These accidents are of so frequent occurrence, compared to dislocation, that the wards of

Guy's Hospital are seldom without an example. The three varieties of this accident already mentioned are—1st, fracture through the neck of the bone, entirely within the capsular ligament; 2dly, at its juncture with the trochanter major, which is external to that ligament; and, 3dly, when the bone is broken through the trochanter major, beyond its junction with the cervix femoris.

It is not our intention to pursue this inquiry step by step: it is needless to say, that the symptoms are accurately described, and the distinctions which mark the nature of the accident are forcibly delineated; we must therefore confine ourselves to such observations as appear to us more particularly tending to confirm the opinions usually entertained on this side of the Channel. It is well known that fractures of the neck of the thigh-bone within the capsular ligament are more frequently met with in women than in men, and that it is an accident confined to an advanced period of life, which the condition of bone in aged persons easily accounts for. Our author denies that this fracture, when transverse, ever unites; and he declares, that all the dissections he made in early life, and the opportunities he has since had of confirming these observations, have fully convinced him that such is the fact. A case in which our author was consulted, and in which the medical attendant had from week to week promised an union of the fracture, leads to an animated appeal to young medical men to *observe*, and not to speculate; and we fully agree with him, "that nothing is *known* in our profession *by guess.*" Indeed, we can scarcely conceive any wider difference than that which exists between guessing and knowing. Firmly, however, as this belief is fixed in Sir Astley's mind, he does not deny the *possibility* of union occurring under certain circumstances; but he is convinced that it must be extremely rare, and he declares that no instance of it has occurred to him.

The causes of this want of union are next scrutinized. The first reason assigned by our author is that, unless bones are not *nearly* in opposition, ossific union is prevented: this he verifies by two instances of fracture, where portions of the tibia were sawn off, and no ossific union took place. He also observed the same result in experiments which he made for this purpose on rabbits; in one of which only one-ninth of an inch of the radius was removed, and the extremities were not united to each other. We were about to quote Mr. Dunn's case as a set-off against these experiments; but, as we find that, in his case, the fibula, though fractured, was not protruded with the tibia,—neither was any portion of it removed,—Sir Astley's conclusions, we conceive, remain still unshaken. The second reason which prevents a boney union, is the want of pressure of one bone on the other, which is increased by the effusion of an additional quantity of fluid within the capsular ligament. But the third reason is, perhaps, the most conclusive of all; and that is, the little action in the head of the thigh-bone when separated from its cervix, its life being then solely supported by the ligamentum teres. In describing the dissection of these cases, we find Sir Astley more than once using the term "serous synovia," to express a thinner species of that fluid than is commonly met with. We have already ventured to protest against the synovial membrane being called a mucous one, and therefore we were grieved to perceive that our author, in this place, has *very nearly* called it a serous one. We are partial to precision in language, and therefore have presumed to notice this additional instance of inadvertence and haste.

Having described the appearances on dissection, our author gives us the result of some experiments on rabbits and dogs, in which he contrived to fracture their thigh-bones within the capsular ligament, and they all confirm the opinion previously delivered; the whole evidence fully establishing, in our mind, the point which Sir Astley has undertaken to prove.

In describing the treatment of this fracture, our author mentions several contrivances that have been adopted, all with the intention of keeping the limb fully extended: the double inclined plane; the plan of suspending a weight to the foot of the fractured side, at the same time taking measures to prevent the body descending in the bed; the extension of both legs, and fastening them securely together at the ankle; and the splint of Boyer, are all mentioned; and, finally, a plan recommended by Mr. Hagedorn is detailed, which Sir Astley mentions as ingenious, but which he thinks will not prevent a displacement of the bone on every motion which the patient is constrained to make for the purpose of evacuating the fæces: he nevertheless, in the spirit of candour that pervades his whole work, recommends a fair trial to be given to it. After all, however, Sir Astley concludes, that all the means he has seen used have proved unavailing. "I have been baffled," he says, "at every attempt to cure, and have not yet witnessed one single example of union in this fracture." With respect to the instances of success that have been published, he is incredulous, because he thinks that the authors are not aware of the distinctive marks of the fracture within the ligament; and which inference he draws from their not mentioning the age, the little shortening of the limb, and the degree of suffering, in their account of these accidents. He can, however, conceive that, if the periosteum covering the neck of the thigh-bone should not be torn through, or that, though the head of the bone be broken, the cervix remains in the acetabulum, that union may be produced; but then he says, that in neither of these rare cases will the limb exhibit the shortened state, which the fracture of the neck of the bone *usually* produces. (p. 143.) There is surely some discrepancy between this last sentence and the preceding paragraphs; for, if union *might be* produced in these two instances, the shortened state of the fractured limb is the only criterion by which that may certainly be known, and which usually (it is not said invariably) takes place, we cannot be justified in neglecting to make the attempt, for a length of time at least sufficient to ensure success, under the possibility of either of the above-mentioned conditions of fracture having existed. "The surgeon," says our author, "must be very careful of the opinion which he gives of the result of these cases. Lameness, in the transverse fracture, is sure to follow; but its degree cannot, at first, be exactly estimated." (p. 144.) It appears that the dissections of several cases of these fractures by Mr. Collis, [Colles,] of Dublin, fully confirm these opinions.

Of fracture of the neck of the thigh-bone without the capsular ligament we shall merely observe, that ossific union may in these cases be expected; and several are detailed, together with the appearances of the bone and joint on dissection. In the treatment of these fractures, the limb is kept in an extended position most perfectly by binding it firmly round the ankle to the sound one, which thus becomes *the splint* to the fractured bone. Various modifications of the double inclined plane have also been employed with success in similar cases, but want of room forbids us to enter into a more minute description of the apparatus.

The fracture through the trochanter major may take place obliquely, without the cervix femoris being at all concerned. The altered position of the trochanter major, and the crepitus upon moving the limb, are the distinguishing marks of this accident, in which ossific union takes place very firmly and quickly. From the detail of the long case communicated by Mr. Harris, of Reading, we find that the fracture of the great trochanter may take place without producing either eversion or inversion of the foot, or shortening of the limb; that the crepitus may also at first remain unnoticed; and that the pain in moving the limb (except across the sound one,) may be but slight. We must here take occasion to observe, that we are sorry that this case had not been curtailed prior to publication: it contains some passages which we do not quite like, and which, however unexceptionable in a private letter from Mr. Harris to Sir Astley Cooper, leave an unpleasant impression upon our minds, which we cannot well express, but which we are confident the attentive reader will understand.

Of fracture just below the trochanter we shall only observe, first, that, if ill-treated, great deformity ensues from the overlapping of the bones, in consequence of the contraction of the iliacus internus and psoas muscles; and that, consequently, the mode of preventing this deformity is to elevate the knee very much over the double inclined plane, and to place the patient nearly in a sitting position: the reasons for which mode of treatment the anatomist will immediately understand and appreciate.

We shall now proceed to the discussion of the chapter on Dislocations of the Ankle-joint, which, on many accounts, we consider one of the most important in the whole work: to arrive at this point, we have passed over upwards of fifty pages rich with a variety of matter on dislocations and fractures of the Knee-joint, Patella, &c.; but, besides that our space is too limited to enter into the consideration of each of these subjects individually, we did not encounter any thing in that portion of the work which called for our especial notice. It must be read, —it should be *studied* by the young surgeon, for it is rich in facts, and full of practical wisdom.

*On Dislocations of the Ankle-joint.*—A concise anatomical description of this joint, together with its ligaments, leads to an enumeration of the different directions in which dislocation may occur in the ankle; three of which only our author has seen, namely, inwards, forwards, and outwards. It is said sometimes to be dislocated backwards; and it has also been thrown upwards between the tibia and fibula. Simple dislocation of the tibia inwards is often connected with fracture of the lower end of the tibia and fibula. In order to distinguish this latter fracture, the leg must be grasped by the hand just above the ankle, and the foot must be freely rotated. In effecting the reduction, let the patient be placed upon the injured side; the leg is to be bent, to relax the muscles; extension made with the foot, in a line with the leg; the surgeon then fixes the thigh, and presses the tibia downwards. Let the leg then be kept on its side in the bent position, with the foot well supported, and a many-tailed bandage applied to keep the parts in their places; two splints, each having a foot-piece, should then be placed on the leg. In the event of inflammation, the usual local and general means of subduing it must be had recourse to. In five or six weeks, the patient may be moved from his bed, and put on crutches; but a much longer time will elapse before he regains the perfect motion of the foot.

We shall pass by the simple dislocation of the tibia forwards, a case by no means unfrequent, in order to notice a partial dislocation of the same kind, which is more rare: in this case, the bone rests half on the os naviculare and half on the astragalus; the fibula is broken; the foot appears but little shortened, nor is there any great projection of the heel. The diagnostic signs are the following: the foot is pointed downwards, and a difficulty is felt in attempting to put it flat to the ground; the heel is drawn up, and the foot is in a great degree immoveable. In a case of this kind, it appears that our author was baffled in his attempts at reduction; and he warns us, in all similar cases, not to rest satisfied until the foot be returned to its natural position, however slight the deviation may at first appear to be. The reduction is effected by the same means as are employed in the complete dislocation forwards.

The luxation of the tibia outwards is the most dangerous of the three; for, in this case, the malleolus internus is obliquely fractured and separated from the bone; the astragalus is also sometimes fractured, and the lower extremity of the fibula is broken into several splinters. In this accident, the proper ligaments of the joints remain untorn, if the fibula is broken; but, if not, they are ruptured; the capsular ligament is torn at its outer part. Reduction is effected "by placing the patient on his back; the thigh is bent at right angles with the body, and the leg at right angles with the thigh; the thigh is then grasped under the ham by one assistant, and the foot by another, whilst the surgeon presses the tibia inwards towards the astragalus." (p. 248.) The position of the limb is to be the same as in simple dislocation. The greatest care must be taken to prevent the foot from being twisted inwards or pointed downwards; and, for this purpose, two splints, with a foot-piece to each and padded, must be applied to the ankle on the outer side of the leg. The severity of this accident calls for more vigorous measures with regard to depletion, as inflammation to a considerable extent may usually be expected to follow its infliction.

*Of compound Dislocations of the Ankle-joint.*—We have already said that, in our estimation, this chapter is the most important one in the whole volume, since it involves a point of practice that has been long and warmly contested, and upon which it is very difficult to speak without saying too little or too much. We have read it over carefully again and again, and we confess the impression that it has left in favour of making the attempt to save the limb in these accidents is stronger than, upon reflection, our calm reason and sober judgment can approve. We do not in the least doubt that Sir Astley Cooper *himself* is perfectly master of all the niceties of each possible case, and that he would decide most judiciously upon any contingency that might arise; but we do not think that he has been altogether happy in placing his subject in a clear point of view, or in dwelling upon those peculiar features of the accident that so often render amputation absolutely necessary; and we should fear that the young surgeon, from the perusal of this chapter, would be led to the almost indiscriminate attempt to save compound dislocations, the encouragement to save so much overbalancing the warning of danger; a mode of practice which we are confident our experienced author had no intention of recommending, without considerable limitation and restriction. Indeed, these limitations are mentioned; but they appear to "halt in the rear" of so many brilliant and extraordinary instances of success, as to be likely altogether to escape the notice of the young and the sanguine. It may be, indeed, that our author thought the young surgeon wanted no spur to perform an operation; and that, therefore, he has made the possibility of dispensing with the knife more prominent than he would otherwise have done.

The first general remark suggested by the perusal of this chapter, is that the whole of the successful cases which he has detailed include nearly every one of the circumstances which he afterwards asserts to be *separately* a substantial reason for amputation: thus, in one we have extensive suppurations;* in another, great deformity of the foot;† in a third, an extensive lacerated wound;‡ and finally, in a fourth, both an advanced period of life and an irritable habit of body.§ The sixteen cases which are published from the correspondence of a number of medical practitioners, in various parts of England, and the nine cases occurring either in his own practice or those of his immediate pupils, have too much the air of being select cases. It is to be observed, that almost all these accidents occurred in young and healthy subjects, with the exception of three; that many of them were boys, or young persons in the prime of life; that extensive *contusion* of the integuments does not appear to have occurred in more than one or two instances; and that, therefore, we cannot be satisfied with the evidence he has adduced, unless it be corroborated by that of the hospital surgeons of the metropolis generally, which we are induced to believe is not so favourable to the plan recommended, but that the failures in the attempt to save limbs so dislocated have been so numerous as to form a very strong argument against the doctrines here delivered. In short, we must warn the young surgeon to recollect, in spite of the high authority of Sir Astley Cooper, (and no one values that authority more than we do,) that, whilst he acknowledges that cases such as he describes have been saved, these results are not of every-day occurrence; and that, before the attempt be made, the age, constitution, habits and situation of life, and the command of proper comforts and attention, must be duly weighed, before a right and sober judgment can be formed as to the possibility of saving a limb. Many

* Case iii. † Case ix. ‡ Case x. § Case xi.

of these reflections, which ought to precede the decision of the question, become in the event prominent, when that event has been fortunate; but how many instances of failure are passed by? how many cases, fatal in their result, but most instructive to the living, are omitted? and which, from their very failure, become interesting, as they tend at once to clear up the difficulty which surrounds this important and much-disputed point.

Let us now leave these general remarks, and resume our analytical labours.

The immediate consequences of the compound dislocation of the ankle-joint, is the exposure of its cavity and the escape of the synovia; inflammation soon becomes established, in which the extremities of the bones and ligaments are equally involved, and suppuration ensues in about five days. Under this process the cartilages become wholly or partially absorbed. This process is attended with severe constitutional irritation, and often lays the foundation for exfoliation of the bones. The granulations arise from the surfaces of the bones and the inner side of the ligament, and thus the intervening cavity becomes filled. Sometimes, says our author, the adhesive process occurs at one part, and the cartilage is not absorbed; whilst granulations are formed at others, where the cartilage was removed by ulceration; and he has seen, after inflammation in the joints, the cartilages remain, and their surfaces adhere. (p. 251.) But permanent anchylosis does not necessarily ensue; for, by employing passive motion as soon as the inflammation has subsided, some degree of motion will be restored: sometimes, indeed, this deficiency in the mobility of the joint is but little apparent. The following circumstances then occur, as necessary consequences of this accident: an extensive suppuration over the joint, with great constitutional derangement; then an ulcerative process, more or less extensive, by which irritative fever is kept up for *a great length of time;* and sometimes, in consequence of ulceration extending to the extremities of the bones, an *additional* constitutional irritation, and protracted disease from exfoliation. After some further discussion upon the causes of the symptoms, our author then proposes his principal question —" Is amputation generally necessary in compound dislocations of the ankle?" His answer is, certainly not. Now, let us contrast this opinion with the following reasons, that he himself declares will give rise to a necessity for amputation in these cases, and then we shall see that this decided negative must be received with much reservation; and that the young surgeon must weigh every one of these circumstances well in his mind, together with all the local and individual peculiarities of the patient, before he can fairly appreciate the force of the precept, which, we do not hesitate to repeat, is both urged too forcibly, and put in a point of view much too prominent and encouraging. The reasons in favour of amputation are—1st, a very extensive lacerated wound; 2dly, the bones being very much shattered; 3dly, it sometimes happens that, when the bone is replaced, it will not remain in its situation, and all the symptoms of the injury become *removed*, (*renewed* is undoubtedly the word intended to be employed, and without which the sentence is unintelligible;) 4th, mortification of the foot; 5th, excessive contusion; 6th, extensive suppuration; 7th, exfoliations of the bone, which, being locked into the surrounding parts of bone, cannot be separated; 8th, excessive deformity of the foot; and, lastly, an irritable state of the constitution.

In the first nine cases recorded by our author, the patients were all in the vigour of life, none exceeding the age of forty-eight. The tenth case is that of an aged man (seventy), intemperate and gouty: the accident was of the worst kind; the articulating surfaces filled with blood and sand, the end of the bone covered with dirt, the man having got up and endeavoured to stand after the accident, and the foot completely turned outwards: in this state he was removed four miles to his residence. This limb was saved, in consequence of the man's refusing to submit to amputation. The case was, within a twelvemonth, brought to so successful a termination, that by the end of that time he could walk without a stick. The remaining cases, with one exception, all relate to young persons; and we pass over the particulars of their treatment, because we shall presently detail the plan recommended by our author to be adopted when it is determined to attempt saving the limb, as including every thing that can be said upon the subject.

From the letters addressed to Sir Astley Cooper upon this subject, we shall venture now to extract a passage or two; and the first that we shall notice is contained in one from Mr. Chandler, of Canterbury. After observing that, in fifteen years, only two accidents of the kind under consideration had occurred, either in his practice or that of his coadjutor, Mr. Fletcher, but which two cases terminated favourably, he goes on to say, "In accomplishing so desirable a point (that of saving the limb), the advantages obtained in a country hospital will, I apprehend, bear a greater proportion in the scale of success, than when the patient is placed in a crowded infirmary of a large manufacturing town, or in the metropolis: the constitution will, in general, be less impaired by excess, poverty, and other circumstances; whilst purity of air in well-ventilated wards materially contributes towards recovery, even if the injury to the joint be extensive; we consequently can be permitted to take greater latitude with our curative means upon an injured joint, relying on the powers of nature, without being under the immediate necessity of anticipating the issue resulting from unfavourable habits, and in situations inimical to disease." (p. 281.)

The next extract we are induced to make is from Mr. HAMMICK's letter, dated from Plymouth, and which our author very truly designates an excellent letter. This gentleman begins by saying that many cases of compound dislocations have fallen under his care and observation, in the course of twenty-four years, and the result of his experience is, that there is not only a chance of saving the limb, but of its being at a future time useful. He very minutely and satisfactorily describes his mode of proceeding where there is a probability of saving the limb, and then continues in these words:—"I have seen more than one case where, after great perseverance and risk, the limb has been saved, but, when the wounds were all healed, found to be of *little or no use.* As an example, a man who had had a compound dislocation of the ankle in the West Indies, from whence he was sent to England as an invalid, became my patient in this hospital, and, when received, (a period of thirteen months from the accident,) had the whole of the lower head of the tibia exposed, black, and carious; which, at the end of a year and a half, came away, more than three inches in length; and, at the end of three years and a half from the injury, he quitted the hospital with the wound healed, but with a shortened, deformed, and anchylosed leg, liable to break out on the slightest injury.—From all I have seen, I should not hesitate to advise the immediate removal of the limb, where the lower heads of the tibia and fibula are very much shattered,—where, together with the compound dislocation of these bones, some of the tarsal bones are displaced and injured; where any large vessels are divided, and cannot be secured without extensive enlargement, and disturbance of the soft parts; where the common integuments, with the neighbouring muscles and tendons, are considerably torn; where the protruded tibia cannot by any means be reduced; where the constitution of the patient is enfeebled at the time of the accident, and not likely to endure pain, discharge, and long confinement."

Having now reversed, in some measure, the arrangement of our author, by pointing out the discouraging circumstances that attend these accidents, and drawn the attention of the young surgeon to those points of the case that demand his peculiar consideration, and which he may be obliged to decide upon in a moment, we shall pursue our course, and describe the treatment of these accidents, being well assured that the vast importance of the subject does not render it necessary for us to apologize for having devoted so large a share of our attention to this discussion.

The mode of reducing the bones differs in no respect from that which has been described in treating of simple dislocations, and, when that is effected, a piece of lint dipped in the patient's blood forms the most natural covering to the wound. A many-tailed bandage, the portions of which should not be sewn together, is then applied: by this plan, any one piece that becomes stiff may be renewed without disturbing the limb. This bandage should always be kept wet with spirits of wine and water. In the inward dislocation, the limb should rest upon its outer side, having on that side a hollow splint applied, with a foot-piece, at right angles; but, in the outward dislocation, it is best to place the limb on the heel, with a splint and foot-piece on each side, and with an aperture in the splint opposite to the wound. In each case the knee should be slightly bent, and great care must be taken to keep the foot at right angles with the leg. The patient should lie on a mattress, and a pillow should extend half-way above the knee, and another rolled under the hip, to support the upper part of the thigh-bone.

The constitutional treatment next becomes a matter of consideration, the necessity for which depends, of course, upon the state and habit of the patient; but, with regard to purgatives, they must be used with great caution, on account of the disturbance they must necessarily occasion to the limb; and our author says he is quite sure that, in compound fracture, he has seen patients destroyed by their administration. The bleeding and purging should be effected as soon as possible after the infliction of the injury, *before* inflammation arises; after which, the liq. ammon. acet. and tinct. opii form the patient's best medicine. After four or five days, if there be much pain in the part, the bandage may be raised to examine the wound; and, if necessary, a corner of the lint may be lifted up to give vent to any matter that may have formed, but this must be done with great circumspection. If, however, adhesion will not take place, then, the lint being removed, simple dressings may be substituted; or, if inflammation runs high, poultices may be applied to the wound, and leeches to the limb; but, as soon as the inflammation is lessened, the poultices should be removed. Sometimes in a few weeks the wound heals, with little suppuration; in other cases, exfoliation retards the cure; and the degree of motion that remains will bear a relation to the quantity of suppuration and ulceration. Three months is, under the most favourable circumstances, the least period that must elapse before the patient can walk with crutches; and according to the extent of the injury, of course, the period will become protracted.

It occasionally has been found necessary, to enable the surgeon more readily to reduce the fractured bone, to saw off the extremity; a practice which Sir Astley Cooper considers may occasionally be adviseable, but on which he remarks, "It is not my intention, however, to advocate either mode of treatment to the exclusion of the other, but to state the reasons which may be justly assigned for the occasional adoption of either." (p. 302.) Our opinion is certainly, upon the whole, favourable to this practice, in conformity with the following reasons which Sir Astley has stated:—1st, it removes the difficulty in reduction; 2dly, if the bone be broken obliquely, by removing the point, it rests without difficulty upon the astragalus; 3dly, it diminishes the spasmodic contractions of the muscles; 4thly, it renders the ulcerative process much less tedious; and, consequently, 5thly, the constitutional irritation is much lessened. These are the principal, but not the whole of the arguments adduced in favour of removing the extremity of the fractured bone, and against which only two objections have been urged: one is, that the limb becomes somewhat shorter, but which our author does not consider of great weight, and we agree with him entirely; and the other consideration is, that the joint becomes necessarily anchylosed, the truth of which is very doubtful; and, indeed, Sir Astley mentions two cases in which this did not happen: and, even if it should, the motion of the tarsal bones becomes so much increased as to be a substitute for that of the ankle.

---

### DIVISION II.

### FOREIGN.

ART. II.—*De l'Hypochondrie et du Suicide, &c. &c.* Par J. P. FALRET, Docteur en Médecine de la Faculté de Paris, &c.—pp. 512. Paris, 1822. [Article on *Hypochondriasis* concluded, from page 77.]

THE indirect occasional causes are principally the abuse of spirituous liquors, tonics, and (according to some) of tea; while many writers mention the immoderate exhibition of purgatives, emetics, and mercurials; but the author before us holds that these "have been placed in the first rank precisely by those physicians who regard the abdomen as the seat of the disease," and, of course, thinks they merit but little notice.

M. Falret then proceeds to a regular analysis of the moral affections incidentally mentioned by M. Villermay as causing hypochondriasis, by which the extent of their influence is unequivocally demonstrated; forty examples of disease from causes purely moral being detailed in a work intended to advocate an opposite opinion. It is, however, but just towards M.

Villermay to remark, that, whatever his opinion may be regarding the seat of the disease, he is fully disposed to allow the importance of moral causes in producing the disease, and modifying the symptoms. "In general," says he, "the moral disorder is sooner displayed: it is better characterized when the hypochondriasis is produced by painful affections of the mind, or by meditations too long continued. On the contrary, when it is the result of a physical cause, the disorder of the organic functions predominates over that of the intellect. In this case, the disease is purely physical; in the other circumstance it is purely moral: that is to say, the alteration of mental faculties is more evident and predominant."*

M. Falret justly regards discovery of the causes of a disease as lending us some assistance in determining its seat, particularly in such a case as the present; for, if it be admitted that hypochondriasis derives its origin from moral influences, it is difficult to avoid the conclusion that they must first operate upon the brain. Here we shall let the author speak for himself, and the following quotation will at once illustrate the views of M. Falret on this disease, and serve as a specimen of the forcible and lively style in which he writes:

"If it be true that the causes of hypochondriasis are intellectual, or moral, it necessarily follows that they must act directly on the brain. Our antagonists, as we have seen, feel all the justice of this inference. However, we should be ill understood if it was supposed that we place in the brain the seat of all those maladies which acknowledge for their cause either painful moral affections, or exertions of the mind too great or too long continued. This would be to place there the seat of the greater number of aneurisms, and many other affections very opposite in their nature. There are many cases where the brain, being strongly constituted, resists the action of the most powerful direct causes: it then reacts on the most irritable organ, or on the one with which it is connected by the closest sympathies, giving rise to occurrences of the most serious nature. Thus, when a disease of the heart, or of another viscus, takes place, as the consequence of a moral cause, its seat is not on that account in the brain; it can only reside in the organ, the function of which is perverted or deranged. Now, under such circumstances, the brain evinces no mark of suffering: if there exist some cerebral symptoms, they are slight transitions, and almost always dependent on the state of the circulation. But can it be denied that the brain may be affected primarily and permanently, when the moral causes are energetic, and when there is a predisposition, either natural or acquired? Can we refuse to recognize the existence of the forms of disorder in an organ submitted to the immediate action of the causes, which is the first to exhibit symptoms of lesion, of which the deranged action is constant, and sometimes proceeds to very considerable extent, even when the

* *Maladies Nerveuses,* 347.

viscera which have been regarded as primarily affected return to their natural state? How can it be maintained that an organ, derangement in the functions of which are indispensably necessary to characterize the disease, are yet not the seat of such disease? I might as well be told, that the spontaneous luxation of the femur (morbus coxarum) resides in the knee, because the pain is often more severe there than in the coxo-femoral articulation. Why, then, have not the phenomena referrible to the head attracted all the attention of medical men?

"I shall here content myself with mentioning two of the principal causes of error in the diagnosis of hypochondriasis: 1st, the opinion of the seat of the passions being in the abdomen; 2d, the habit, long prevalent among physicians, of regarding the brain as only secondarily affected.

"Does the tongue become yellow, the epigastric region painful, or are nausea or vomiting excited, under the influence of an intellectual or moral cause, immediately some exclaim, there is gastric derangement, and others gastric irritation, or even gastritis. But why refuse to take into consideration the nature of the cause? why not give their just value to the concomitant phenomena,—such as the head-ach, a certain torpor of ideas, (if I may thus express myself,) the insomnolence, the starting awake, the disorder of the cerebral circulation, the irregularity of the animal heat, the constraint of the voluntary motions? I believe, in the existence of gastritis, when the cerebral symptoms having ceased, the disorder of the stomach continues, or increases in degree; but, in the opposite case, is it reasonable not to admit the cerebral irritation as the cause, and the irritation of the stomach as the effect? Ought the diagnosis to be taken from symptoms of small importance and of ephemeral duration, which are often wanting, while the nature of the disease rests always the same? This, however, has been done with regard to hypochondriasis." (p. 402—5.)

*Symptoms.*—Physicians, entertaining a different opinion regarding the seat of the complaint, having also given a different history of the symptoms; M. Villermay and others asserting that disorder of the digestive functions mark the first stages, and that the extension of the disease to the organs of the chest constitutes the second; the third being marked by the lesion of the organs of animal life. "Soon (we translate the words of M. Villermay,) the organs of our external relations, or which place us in relation with all which surrounds us, participate in the trouble of the sensitive or interior life."

This description M. Falret confidently pronounces to be derived from other sources than actual observation; and, according to this author, the train of symptoms is in reality directly the reverse of what is stated in the most esteemed works. He enumerates shortly the phenomena attending the senses, from which it appears that the sight, hearing, smell, and taste, are subject to painful or depraved affections, and sometimes endowed with a morbid state of sensibility: this is particularly the case with regard to the touch,—the slightest degree either of heat or cold producing strong impressions. There is occasionally an approach to syncope; during which, however, the patient does not entirely lose his consciousness, and from which he speedily recovers. Pain in the head is likewise a general occurrence, which varies in situation and in degree: in some the integuments become preternaturally tender, and the patient even complains of exquisite pain in the hair.

We come now to the mental functions. The intellect does not, in general, suffer much at the commencement, and sometimes it even appears more vivid than usual, though this does not last long. The memory becomes impaired, and the judgment slow, but sound, "except in what regards the health." "The imagination is very active, and very mobile: in the last stage of hypochondriasis, the disorder of the intellect often constitutes true mental alienation." The whims and fantasies of hypochondriacs are very numerous, and many of them such as to provoke a smile, even when we pity the subject of such strange delusions. M. Falret mentions, as not uncommon, the idea of a detonation, which they compare to the discharge of a piece of fire-arms, taking place in the head, breast, or belly; while others imagine that they feel the movements of some living creature in one or more parts of the body. Our author knew a lady who, when she looked at her skin, thought it scaly like that of a carp, but she could immediately rectify this false impression by the sense of touch. To these instances mentioned by M. Falret, we may add a few taken from other sources.—"GREDING gives an account of a medical practitioner who applied to him for assistance, under an impression that his stomach was filled with frogs, which had been successively spawning ever since he had bathed, when a boy, in a pool, in which he perceived a few tadpoles. He had spent his life in trying to expel this imaginary evil, and had travelled to numerous places to consult the first physicians of the day upon his obstinate malady. It was in vain to attempt convincing him that the gurglings, or borborygmi, he heard, were from extricated and erratic wind. He argued himself, says M. Greding, into a great passion in my presence, and then asked me if I did not hear the frogs croak."* The learned writer from whom we have taken the above quotation likewise informs us, that MARCELLUS DONATUS mentions a baker of Ferara, who imagined himself a lump of butter, and durst not sit in the sun or near a fire, for fear of being melted. ZIMMERMAN met with a case where a man fancied himself a barley-corn, and did not venture to go out, lest he should be picked up by some bird.

* GOOD's *Study of Medicine*, vol. iii. p. 147.

And it occurred to ourselves to know of a gentleman who supposed his "nether bulk" to be made of glass, and who never sat down without much caution. M. Villermay mentions a hypochondriac who had set apart one of his rooms for his chamber-pots, of which he had made a very numerous collection filled with urine. He made use of a new one every day, and frequently passed them all in review, forming a kind of museum new in the history of natural curiosities.

One of the most annoying absurdities among the symptoms of hypochondriasis is the degree of vacillation in every purpose, and the deliberation which frequently precedes the most trivial actions. Dr. Reid, in his amusing work upon Nervous Diseases, mentions that he called upon a young gentleman at Oxford, who had injured his health by severe application. It was afternoon, and his friend was still in bed, not having been able to determine whether he should put on his smallclothes or a pair of pantaloons. Having pursued his ratiocination for some time longer, the important decision was made in favour of the latter; but he had not been dressed many minutes before he repented of his choice, and during the rest of the day he wore breeches. We once lodged at the house of a worthy old lady, who, though rather infirm, frequently took the trouble of climbing up stairs to request our opinion whether she ought to rest herself by sitting or reclining on her sofa. From these and similar instances we recognize the fidelity of the picture of an hypochondriac as given by Moliere, in his "Malade Imaginaire," when he makes M. Argan say, "Monsieur Purgon, m'a dit de me promener le matin dans ma chambre douze allées et douze venues, mais j'ai oublié à lui demander si c'est en long ou en large."

In all such examples as those enumerated above, the affinity to derangement is very striking: tormented by the dread of imaginary evils, their time is spent in gloomy forebodings of the future, or the most minute attention to the state of their health, harassing to themselves and tiresome to others. "Overwhelmed with their melancholy situation, they sometimes have recourse to suicide." It is the consideration of this circumstance, as we have before mentioned, which has induced us to reverse the order of M. Falret.

The author next proceeds to the "sympathetic phenomena," consisting in those lesions which most writers have supposed to be primary, but which he thinks are secondary, both in occurrence and importance. The first class of these symptoms regards the digestive functions, which, as every one knows, becomes more or less impaired; but, as the remarks of our author do not appear to us to contain any thing new, we shall pass on to the sanguiferous system, which is, next to digestion, the most frequently deranged. The state of the circulation in the brain is considered at some length, and is shown to be irregular, frequently attended with pulsation so strong as to be counted by the patient, and giving rise to flushing of the face, uneasy sensations within the head, and insomnolence. M. Falret has remarked that, in hypochondriacs, palpitations of the heart often take place to a great extent, particularly on going to sleep, but that they have intermissions, and are diminished during moderate exercise. Sometimes one or two pulsations of the radial artery are lost on either side, and occasionally on both sides at once: in other instances, the whole arterial system seems overturned in its functions, (*bouleversé dans ses fonctions*,) of which the author gives an example from experience in his own person. "The pulsations of all my arteries, which I heard very distinctly, were so strong, that I dared not make the least movement, so much was I afraid of their bursting. This took place principally at night, at the moment of going to bed. My anxiety was extreme. Happily this revolution was not of long continuance, and a profound sleep freed me from this state of agony, which I know not how to describe." (p. 427.)

Cough, oppression about the chest, or a sense of constriction about the larynx, are described as affecting the respiratory system, which, as well as all the other symptoms above mentioned, are regarded as *nervous*, but dependent on disease of the brain.

*Analysis of Cases.*—In illustration of his doctrines, the author proceeds to the detail of cases, not from his own observations, although his experience under Pinel and Esquirol must have furnished him with many, but taken from the writings of M. Villermay, and others maintaining views opposite to his own, "Forcé donc d'opter entre les facts des autres et les miens propres, je n'ai pas balancé à donner la préférence à ceux de mes adversaires; les miens auraient pu paraitre suspects à des esprits sévères."

Twelve cases are related, from which, of course, he draws conclusions favourable to his own views: of these we can give one only, being warned by every line to be brief; it will show sufficiently well the manner in which our author explains the symptoms.*

"A chemist, aged twenty-two years, of bilious temperament, having experienced much vexation, was taken, without any other known cause, with irregular palpitations in the region of the heart, and with extreme oppression of breathing: the violence of these symptoms obliged him to become a patient at La Charité. At this time his countenance was thin, with little colour, and sallow; his eyes appeared weak;

* The case is from the work of CORVISART; the *reflections* are by M. FALRET.

the tongue yellowish at the root; the respiration very difficult. He experienced frequent and severe palpitations, or felt violent and irregular beating in the region of the heart. The pulse was hard and vibrating, but sufficiently regular: the chest, however, sounded well on percussion. The right hypochondrium was a little painful. The suspicion which I at first conceived of organic lesion of the heart, gave place to the hope that a simple spasmodic affection might give rise to the series of morbid phenomena. The recent date of the disease (five months, however,)—the tenderness in the region of the liver,—the yellowish tinge which pervaded the surface, contributed not a little to confirm this idea; and, from the second day of his admission into the hospital, I directed the treatment towards the object in view. I prescribed diluting drinks, antispasmodics, and baths. The patient was bled once, about the fourth day. I endeavoured by reasoning to dissipate his imaginary chagrin. In a short time I had the satisfaction to see that these means, far from being ineffectual, as might have been apprehended, produced every day more decided improvement. The cure was so quick that, fifteen days after his admission, the patient left the hospital, enjoying a state of health which did not appear likely to be soon deranged.

"May we not regard this affection as a kind of nervous hypochondriasis, caused by the state of the liver, and subsequently giving rise to the symptoms of irregular action of the heart and oppressed respiration.

"*Reflections.*—It is impossible not to admit, with the celebrated Corvisart, that this person was affected with a species of hypochondriasis, or rather with real hypochondriasis; but then where was the seat of this affection? The knowledge of the cause alone ought to make us presuppose that the brain was primarily concerned: chagrin could not act immediately either on the heart or liver; these organs could only experience its influence through the medium of the brain and nervous system. The observation is deficient in details relative to the cerebral symptoms, but we know had *imaginary* chagrins; that is to say, that he experienced false sensations: in a word, that his intellect was not sound. Finally, the treatment throws great light on the seat of hypochondriasis, since it was successfully directed towards the brain. * * * How, then, does it happen that Corvisart regards the liver as the primary source of the disease, and considers the disorders in the functions of the heart and lungs as consecutive? The brain was not suspected of being affected, even secondarily. Is it not, however, in conformity with the general principles of pathology to place the seat of an affection in the organ which has received the first impression of the occasional cause, and which has always afforded unanswerable proof of being deranged in its functions? How attribute all this disorder to an organ which did not appear diseased till after the lesion of three principal organs—the brain, the heart, and the lungs; and which must necessarily have been but slightly affected, since, in the treatment, it did not occupy the attention of so distinguished a practitioner. Such is the effect of preconceived ideas upon the best heads?"

From the details it appears that the progress, complications, and terminations of hypochondriasis, are very various, and require caution in the prognosis. BAGLIVI gives a very favourable view of it; TISSOT one very much the reverse; and Falret adopts the golden medium, tempering the "sanari facile solent" of the one, with the "morbus profecto rebellis et vix curationis capax" of the other.

*Examinations after death.*—These have exhibited every variety of morbid appearance, but without any such regularity as to lead to general conclusions: indeed, our readers must before this time have discovered that M. Falret endeavours to prove the brain to be the seat of disease,—not by demonstration, but by pathological reasoning, arguing "that it is extremely difficult to establish a line of demarcation between the sound and morbid conditions of an organ so little known in its structure and mode of action; and that an examination of the symptoms, and the order of their succession, is sufficient to determine the seat of a disease." Nevertheless, three cases of hypochondriasis are detailed,—one from Morgagni, one from Villermay, and the third from his own observation; in all of which effusion was found in the brain. The number of facts, however, is too inconsiderable to add any important assistance to the arguments of the author.

*Diagnosis.*—This is dispatched in a very cursory manner, and our account of it shall be still more laconic. M. Falret regards the distinguishing symptom of hypochondriasis as consisting in the mental affection; he considers that indigestion, or any other symptom except this, can take place without hypochondriasis; but that cases of the latter are found in which the mind alone is diseased.

In the *treatment*, the author takes his indications from the pathological views on which the whole doctrine of his essay rests. The first object is to gain the confidence of the patient, so as to obtain influence over his mind. This is best effected by entering into his feelings, listening to the history of his complaints, and giving minute attention to the symptoms; and, above all, we are enjoined to avoid any expression which might lead him to suppose either that his disease is imaginary or allied to mental alienation. Exercise, amusement, and cheerful society, added to change of occupation and scene, are among the principal therapeutical means to be employed, and certainly surpass in efficacy the whole range of the materia medica. To these our author suggests the propriety of adding the occasional use of leeches behind the ears, and cold applications to the head. With regard to the sympathetic disorders, the injunction is very laconic,—*subtata causa, tollitur effectus.* We here anticipate the exclamation of our readers,—What, then, after all, M. Falret has written a long essay to show that the seat of hypochondriasis is in the brain, without giving dissections; and ends by avowing that the treatment consists in doing nothing!

That similar objections have been made on the other side of the Channel, is obvious from the following passage:—"What! you count for nothing having directed the treatment towards the organ which suffers,—the seat of the whole disorder? Besides, I do not pretend that we are to abstain from every kind of medicine; but I believe that I have demonstrated the necessity of having always in view that the brain is the seat of hypochondriasis, and of proscribing the excitement of other organs, because their affections are secondary."

In conclusion we would remark, that if M. Falret has failed to convince us that the sources of hypochondriasis are to be found exclusively in the brain, we still think his essay goes far to prove that it does not primarily originate in the abdominal viscera, according to the popular opinion. Yet the author seems to have forgot that the absence of disease in the belly is but a negative proof of its existence in the head; and it would not be difficult, if we chose to select the nerves supplying the organs of digestion, to show that the history of the symptoms might be accounted for as well by disorder in their functions as in the brain itself. The style of M. Falret is lively, his illustrations are good, and his pathology, if sometimes carried rather farther than we are disposed to follow him, displays much research and ingenuity. In the treatment, we think he regards the exhibition of medicines too little: not that we place much confidence in their effects, or mean to approve the routine practice of daily drenching hypochondriacs with a certain quantity of drugs, but because the love of swallowing physic constitutes part of the disease. We think a practitioner, from the perusal of M. Falret's book, would be rather apt to prohibit this indulgence, and, in such case, would probably not long retain his patient: in this dilemma, we recommend to the attention of our readers a specific which we have long been in the habit of giving with great success,—we mean bread-pills.

